# Mental health on two continua: mental wellbeing and common mental disorders in a community-based cross-sectional study with women in urban informal settlements in India

**DOI:** 10.1186/s12905-024-03389-1

**Published:** 2024-10-09

**Authors:** Suman Kanougiya, Nayreen Daruwalla, David Osrin

**Affiliations:** 1 School of Public Health, SRM Institute of Science and Technology, Kattankulathur, Chennai, Tamil Nadu 603203 India; 2 Program on Prevention of Violence Against Women and Children, SNEHA, Mumbai, Maharashtra 400017 India; 3 Institute for Global Health, University College London, London, WC1N IEH UK

**Keywords:** Mental Health, Psychological well-being, Depression, Anxiety, Poverty areas, India.

## Abstract

**Background:**

We considered the suggestion that mental health is the product of two intersecting continua: psychological distress and mental wellbeing.

**Objectives:**

To understand prevalences of low mental wellbeing, depression and anxiety, and examine associations between them and with sociodemographic variables.

**Methods:**

In a cross-sectional survey in informal settlements, 4906 women aged 18–49 years answered questions on mental wellbeing (Short Warwick-Edinburgh Mental Wellbeing Scale: SWEMWBS) and symptoms of depression (Patient Health Questionnaire: PHQ-9) and anxiety (Generalised Anxiety Disorder: GAD-7). We used regression models to examine associations of lower mental wellbeing with symptoms suggesting moderate-to-severe depression and anxiety, and with sociodemographic characteristics.

**Results:**

About 15% of women reported symptoms of low wellbeing, 9% symptoms of moderate-to-severe depression, and 6% symptoms of moderate-to-severe anxiety. Women with low wellbeing did not necessarily report symptoms suggesting anxiety or depression, and women with anxiety or depression did not necessarily report low wellbeing. In adjusted models, poorer and less educated women were more likely to report low wellbeing. Symptoms of anxiety or depression were more likely to be reported by widowed, separated, or divorced women, women who were in paid employment, and women who used drugs or alcohol themselves or whose partners did. Women with low wellbeing had at least double the odds of reporting symptoms of moderate-to-severe depression or anxiety than women who reported greater wellbeing.

**Conclusion:**

The findings support the idea of two continua of mental health. How individual women cope with mental illness and nevertheless enjoy a state of wellbeing deserves more study.

**Supplementary Information:**

The online version contains supplementary material available at 10.1186/s12905-024-03389-1.

**Table:** 

**Text box 1. Summary box**	
**What is already known on this topic?**	
• It has been suggested that mental health has two dimensions: psychological distress (corresponding with mental illness) and mental wellbeing (corresponding with feeling good and functioning well). There is some supportive evidence for this idea, although little from the global South.	
**What this study adds**	
• The study is the first to use question scales to ask women in informal settlements in India about their mental wellbeing, depression and anxiety. Almost a quarter of women reported symptoms suggesting low mental wellbeing, depression or anxiety. Substantial numbers reported low mental wellbeing in the absence of psychological distress, and some felt good and functioned well in the presence of psychological distress.	
**How this study might affect research**,** practice or policy**	
• Understanding of the dimensions of mental health in informal settlements in which people are exposed to multiple stressors is limited. The idea of two continua of mental health merits study, particularly in terms of its implications for services. The finding that some women are able to maintain a sense of mental wellbeing despite psychological distress has implications for research on coping abilities.	

## Background

Recent estimates put the global burden of mental illness at around 420 million disability-adjusted life years, accounting for one in every six years lived with disability and an economic cost of USD 5 trillion [1, 2]. Before the Covid-19 pandemic began, an estimated 970 million people were living with a mental disorder. More than half were women (508 million), around one-third had an anxiety disorder (301 million) [2], and 82% of them lived in a low- or middle-income country [2]. Stark though these figures are, they focus on mental illness rather than mental health. The World Health Organization (WHO) defines mental health as “a state of well-being that enables people to cope with the stresses of life, realize their abilities, learn well and work well, and contribute to their community” [2]. This is in keeping with the idea of health as more than the absence of disease, a holistic framing that predates its articulation by philosophers such as Aristotle and position statements such as the Alma Ata Declaration of 1978 [3].

Although a focus on mental illness characterizes much of the discussion among policymakers, clinicians and researchers, it has been suggested that mental health has two dimensions: psychological distress and mental wellbeing. The idea is implicit in the WHO characterization of mental health as a continuum state with experiences ranging from an optimal state of wellbeing to debilitating states of great suffering and emotional pain [2, 3]. Linked most with the work of Keyes, the two continua model suggests that mental health is located in a field defined by intersecting axes: a continuum of mental wellbeing and a continuum of mental illness [4, 5].

Mental wellbeing is a combination of optimal functioning and subjective emotional experiences [3, 6, 7]. Its components include individual potential, meaningfulness, good relationships, sense of purpose and the value of one’s life [6, 8]. Wellbeing has itself been theoretically divided into feeling good and functioning well [9]: hedonic wellbeing (pleasure, happiness, life satisfaction) [10], and eudemonic wellbeing (good relationships, fulfilment, self-realisation) [4, 6, 9, 11]. A person can be thought of as flourishing when they both feel good and function well. Wellbeing is linked with achievement in personal, interpersonal and professional spheres and is important for creating and maintaining healthy societies [12]. It is linked with better physical health and longevity [13], work performance, life satisfaction, and economic and social development [14–17]. People with greater wellbeing tend to be more productive at work, learn better and be creative and more engaged in prosocial behaviour and favourable interpersonal connection [18]. People with lower wellbeing use mental health services more frequently [19], are more likely to report symptoms of depression [20, 21], suffer depression of greater severity, and are more likely to self-harm [22, 23]. These associations may be stronger than those described for social determinants of mental health such as socioeconomic status, human capital, working conditions, or family background [24].

In India, estimates of the prevalence of mental illness range from 2 to 57% in the general population [25–27] and 8–61% in urban informal settlements [28–30]. Analogous estimates are not available for wellbeing, although subjective wellbeing on a five-question scale was associated with neighbourhood cohesion in two informal settlements in Delhi [31]. Despite progress in physical healthcare infrastructure, the Indian mental healthcare system faces numerous challenges: shortages of clinicians at all levels, fragmented services, insufficient financial support and resources, social stigma and discrimination, perceived coercion, and limited awareness [3, 26, 32–35]. Mental health receives 0.16% of the national healthcare budget, itself 3.2% of public sector expenditure. India’s 2017 Mental Healthcare Act (MHCA, 2017) represents a significant advance in establishing a legal framework for mental health and the decriminalization of self-harming behaviors, including suicide. It provides direction for services for people living with mental illness and aims to protect, promote and fulfil their rights to convenient, quality, affordable and accessible mental healthcare [36]. It does not explicitly address wellbeing.

### Objective

The non-government organization SNEHA (Society for Nutrition, Education and Health Action) has been implementing a programme on prevention of violence against women in Mumbai’s informal settlements for the past two decades. This group of socioeconomically and environmentally disadvantaged women in urban India are under considerable pressure and their concerns may be rather different from those of the Western, Educated, Industrialized, Rich, and Democratic (WEIRD) participants who characterize the literature [37]. Central to our activities is the need to integrate support for women’s mental health in the response to violence, and this has led us to consider the use of assessment tools for common mental disorders such as depression and anxiety.

The importance of social determinants as causes and triggers for both violence and common mental disorders is manifest daily in the accounts of our clients and participants. Structural conditions such as income and socioeconomic position, education, employment, housing and neighbourhood conditions, food security, childhood adversity, discrimination, and access to affordable healthcare all have the potential to affect mental health outcomes, contribute to mental health disparities, and promote positive mental health [35, 38, 39]. The intersection of poverty, inequalities, distress and struggle—of the psychological with the sociostructural—supports a composite view of mental health in terms of psychological distress and mental wellbeing. It also speaks to contemporary discussions in global mental health [40], and particularly the issue of intersection between multiple dimensions of experience and need [41, 42]. For this reason, we included measures of both psychological distress and mental wellbeing in our assessment tools. Our objectives were (1) to compare the findings of scales for symptoms of depression and anxiety with a scale for mental wellbeing, and (2) to examine associations with sociodemographic characteristics.

## Methods

### Setting, design, and participants

Residents of informal settlements face more challenges to their mental health than non-slum and rural residents [43–46]. High population density, poor housing and substandard living conditions exacerbate risks of injury and infectious diseases and probably increase the risk of common mental disorders [43–45, 47]. The study used data collected before the SNEHA TARA (Taking Action Reaching All) cluster randomized controlled trial in Mumbai, India [48]. In a cross-sectional systematic random sample survey, we interviewed around 100 women in each of 50 clusters of approximately 500 households. Between 5th December 2017 and March 2019, 5277 households were approached for the survey. A sample of 4900 from an approximate population of 125,000 would have a precision of approximately 1%, and would have 80% power to detect a difference of 6% in prevalence estimates for domestic violence of 10–20%. The survey respondents were women aged 18 to 49 years.

### Data collection

After a three-month training program, a group of 16 women interviewers mapped study areas and residences and developed a list of homes with potential respondents. Starting at a random point in each cluster, interviewers approached one woman aged 18–49 years in every second home on the list. Each cluster yielded data from 100 women. Interviews were prearranged to ensure confidentiality and privacy. Following the provision of information regarding the aim of the interview and their right to withdraw, participants provided signed consent. Interviewers used Android tablets to input data into a database within the CommCare platform (www.dimagi.com/commcare/). Details are available elsewhere [49]. Data were collected in accordance with the General Data Protection Regulation 2018 and stored in a MySQL database overseen by SNEHA information managers.

We had a duty of care if respondents disclosed violence or mental health concerns. We established a network with local police stations and social services in advance of the survey to facilitate referral and service linkages for women who needed them. Interviewers followed support protocols which included safety assessment, crisis intervention, home visits, counselling, liaison with healthcare, police, and legal services, and developing safety and follow-up plans for the survivor and her family. Three field supervisors supported interviewers by providing a direct connection to counselling services by phone at any time. Investigators also received automated notification of the need for referral if a respondent’s score on mental health screens was suggestive of concern. Participants were given a visiting card that was easy to hide and contained addresses and phone numbers of essential services, along with a toll-free helpline number. SNEHA counselors and community networks ensured follow-up [48].

### Outcome variables: wellbeing and symptoms of anxiety and depression

To examine associations with variables such as spousal characteristics, we limited the dataset to ever-married women. We examined self-reported mental wellbeing and mental illness. The Short Warwick-Edinburgh Mental Wellbeing Scale-7 (SWEMWBS) assessed participants’ mental wellbeing in the past two weeks. The SWEMWBS is psychometrically robust, is suited for population use with individuals aged 13 years or older, and has been used to evaluate wellbeing interventions in demographic research worldwide, including the general population, older people, stigmatised minorities, people living with schizophrenia, and service users with mental health concerns [50–52]. The Hindi version has been validated [53–55]. The Patient Health Questionnaire (PHQ-9) was administered to screen for depression [56] and the Generalized Anxiety Disorder scale (GAD-7) for anxiety in the last two weeks [57]. Both have been validated in India and, in both cases, a score of > = 10 suggests clinically significant problems requiring further examination [58].

### Exposure variables: demographic and socioeconomic characteristics

We included marital status, age, education, employment, substance use, faith, caste and socioeconomic position as potential risk factors for low wellbeing, depression and anxiety based on prior research in India [59–64]. Socioeconomic position was derived from standardized weights for the first component of a principal components analysis of household assets [65, 66].

### Statistical analysis

We checked the reliability of the SWEMWBS, PHQ-9, and GAD-7 with Cronbach’s alpha. We used a score of > = 10 on the PHQ-9 and GAD-7 to indicate moderate-to-severe symptoms of depression or anxiety [58]. With a mean SWEMWBS score of 24.07 and a standard deviation of 5.63, 15% of the sample would be predicted to have a score of > = 29.70 (= 24.07 + 5.63) or < = 18.44 (= 24.07–5.63). We established a cut-off score of < = 18.44 as an indication of low mental wellbeing [67]. We tabulated frequencies and proportions of women who screened positive for low mental wellbeing, and positive for moderate-to severe depression on the PHQ-9 and moderate-to-severe anxiety on the GAD-7. We developed unadjusted and adjusted logistic regression models for low wellbeing, moderate-to-severe depressive symptoms, and moderate-to-severe anxiety symptoms to investigate their association with the following exposure variables: marital status, age, education, faith, caste, socioeconomic asset quintile, employment of women and their husbands, and alcohol or drug use by women or their husbands, suggested as potential variables affecting wellbeing and mental health [68]. We examined the association between low wellbeing as an independent variable and moderate-to-severe depression and anxiety as dependent variables in unadjusted and adjusted logistic regression models. Models were adjusted for background characteristics as described above. We also computed unadjusted and adjusted linear regression models for wellbeing, depression, and anxiety scores. We used survey commands in STATA 15.0 (StataCorp LLC) for all analyses.

## Results

### Participant characteristics

Table 1 summarises the characteristics of 4906 women aged 18 to 49 years who had been married at least once and were interviewed between December 2017 and March 2019. 42% had secondary or higher education, while only a quarter were engaged in paid work, mostly in the informal sector or as domestic workers, and earned a mean USD 163 a year. Most households were of substantial construction, but did not have their own toilets.

**Table 1:** Characteristics of 4906 ever-married women respondents in informal settlements in Mumbai, India

Characteristic	*n*	(%)
**Marital status**		
Currently married	4694	(96.0)
Widowed, separated or divorced	212	(4.0)
***Age in completed years***		
18–19 y	57	(1.2)
20–29 y	1911	(39.0)
30–39 y	2031	(41.4)
40–49 y	907	(18.5)
***Education in completed years***		
No formal education	938	(19.0)
Primary 1–5 y	846	(17.0)
Middle 6–8 y	1099	(22.0)
Secondary 9–10 y	1105	(23.0)
Higher secondary 11–12 y	533	(11.0)
Higher 12 y or more	385	(8.0)
***Respondent employed***	1182	(24.0)
***Respondent monthly income***,*** INR***		
< 1000	233	(20.0)
1000–2999	303	(27.0)
3000–5999	279	(25.0)
6000+	322	(28.0)
***Respondent uses alcohol or drugs***	612	(12.0)
***Husband age in completed years***		
18–19 y	14	(< 1)
20–29 y	917	(19.0)
30–39 y	2102	(44.0)
40–49 y	1370	(29.0)
50 + y	391	(8.0)
***Husband employed***	4686	(98.0)
***Husband monthly income***,*** INR***		
< 10,000	1095	(23.0)
10,000–11,999	997	(21.0)
12,000–14,999	652	(14.0)
15,000+	1942	(41.0)
***Husband uses alcohol or drugs***	2100	(44.0)
***Housing fabric***		
Insubstantial	336	(7.0)
Mixed	2052	(42.0)
Robust	2518	(51.0)
***Toilet facility type***		
Private	836	(17.0)
Public	4368	(82.0)
No facility	2	(< 1)
***Faith***		
Hindu	1826	(37.0)
Muslim	2882	(59.0)
Other	198	(4.0)
***Caste***		
General	2854	(58.0)
Other Backward Caste	1180	(24.0)
Scheduled Tribe or Caste	872	(18.0)
***Socioeconomic quintile***		
1 poorest	969	(21.0)
2	936	(20.0)
3	934	(20.0)
4	933	(20.0)
5 least poor	935	(20.0)

### Characteristics of 4906 ever-married women respondents in informal settlements in reported wellbeing and symptoms of anxiety and depression

Cronbach’s alpha indicated acceptable internal consistency for the SWEMWBS (α 0.737), PHQ-9 (α 0.860), and GAD-7 (α 0.843). SWEMWBS scores correlated negatively with PHQ-9 (r -0.206; *p* < 0.001) and GAD-7 scores (r -0.199; <0.001). There was a positive correlation between PHQ-9 and GAD-7 scores (r 0.789; *p* < 0.001). On self-report, 15% of women had symptoms suggesting low wellbeing, 9% symptoms suggestive of moderate-to-severe depression, and 6% symptoms suggestive of moderate-to-severe anxiety. Almost 23% reported either low wellbeing or symptoms suggesting moderate-to-severe depression or anxiety.

**Fig. 1:**
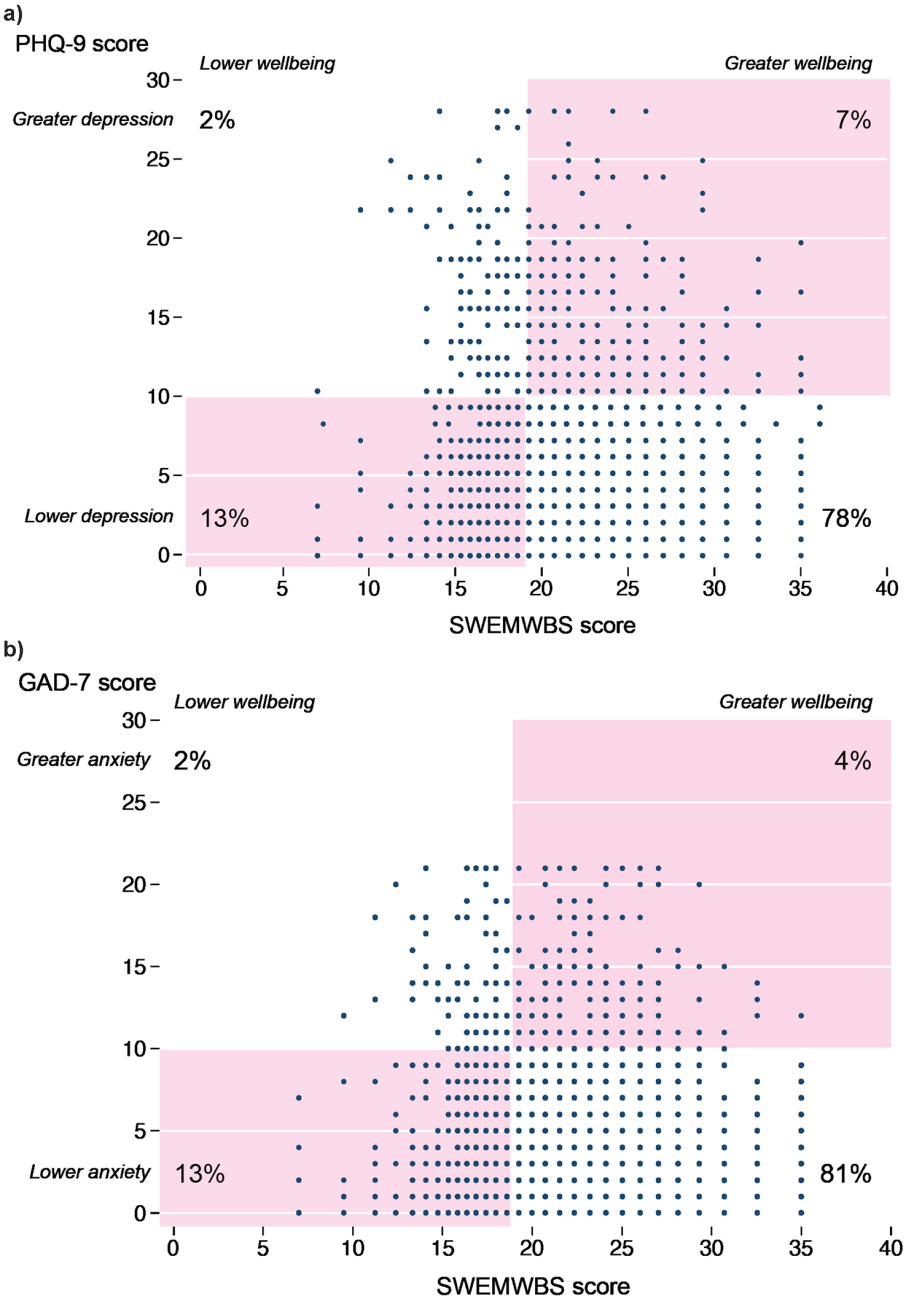
Self-reported symptoms suggesting common mental disorder, by symptoms suggesting lower or greater wellbeing on Short Warwick-Edinburgh Mental Wellbeing Scale (SWEMWBS): **(a)** Patient Health Questionnaire 9 (PHQ-9); **(b)** Generalised Anxiety Disorder 7 (GAD-7)

Figure 1 plots PHQ-9 scores and GAD-7 scores against SWEMWBS scores. The plots are divided into quadrants by lines corresponding with cut-offs suggesting clinical concern. The largest numbers of individuals fell into the quadrant corresponding to acceptable wellbeing and no concern about anxiety or depression. Although 2% of women reported symptoms suggesting depression and 2% anxiety in the presence of low wellbeing, there were two mismatched quadrants. 13% reported symptoms of low wellbeing without depression or anxiety, 7% reported symptoms of depression, and 4% reported symptoms suggestive of anxiety, without obviously low wellbeing.

Table 2 summarises the prevalence of low wellbeing and moderate-to-severe depression and anxiety, categorised by women’s background characteristics.

**Table 2:** Screens for low wellbeing and symptoms of moderate-to-severe depression and anxiety, by characteristics of 4906 ever-married women in urban informal settlements, Mumbai, India

	Low wellbeing on SWEMWBS				Moderate-to-severe depression symptoms on PHQ-9				Moderate-to-severe anxiety symptoms on GAD-7			
Characteristic	No		Yes		No		Yes		No		Yes	
	*n*	(%)	*n*	(%)	*n*	(%)	*n*	(%)	*n*	(%)	*n*	(%)
***Marital status***												
Currently married	3999	(85.2)	695	(14.8)	371	(92.1)	4694	(7.9)	4443	(94.7)	251	(5.4)
Widowed, separated, divorced	175	(82.6)	37	(17.5)	140	(66.0)	72	(34.0)	164	(77.4)	48	(22.6)
***Age (years)***												
18–19	48	(84.2)	9	(15.8)	52	(91.2)	5	(8.8)	53	(93.0)	4	(7.0)
20–29	1605	(84.0)	306	(16.0)	1757	(91.9)	154	(8.1)	1813	(94.9)	98	(5.1)
30–39	1732	(85.3)	299	(14.7)	1863	(91.7)	168	(8.3)	1919	(94.5)	112	(5.5)
40–49	789	(87.0)	118	(13.0)	791	(87.2)	116	(12.8)	822	(90.6)	85	(9.4)
***Schooling***												
No schooling	763	(81.3)	175	(18.7)	109	(88.4)	938	(11.6)	865	(92.2)	73	(7.8)
Primary, class 1–5	687	(81.2)	159	(18.8)	760	(89.8)	86	(10.2)	789	(93.3)	57	(6.7)
Middle, class 6–8	933	(84.9)	166	(15.1)	981	(89.3)	118	(10.7)	1015	(92.4)	84	(7.6)
Secondary, class 9–10	970	(87.8)	135	(12.2)	1027	(92.9)	78	(7.1)	1055	(95.5)	50	(4.5)
Higher secondary, class 11–12	475	(89.1)	58	(10.9)	499	(93.6)	34	(6.4)	510	(95.7)	23	(4.3)
Higher education	346	(89.9)	39	(10.1)	367	(95.3)	18	(4.7)	373	(96.9)	12	(3.1)
***Employment of respondent***												
No remunerated employment	3153	(84.7)	571	(15.3)	3445	(92.5)	279	(7.5)	3536	(95.0)	188	(5.1)
In remunerated employment	1021	(86.4)	161	(13.6)	1018	(86.1)	164	(13.9)	1071	(90.6)	111	(9.4)
***Employment of husband***												
No remunerated employment	79	(79.8)	20	(20.2)	74	(74.8)	25	(25.3)	85	(85.9)	14	(14.1)
In remunerated employment	3993	(85.2)	693	(14.8)	4305	(91.9)	381	(8.1)	4426	(94.5)	260	(5.6)
***Alcohol or drug use by respondent***												
No	3647	(84.9)	647	(15.1)	3950	(92.0)	344	(8.0)	4064	(94.6)	230	(5.4)
Yes	527	(86.1)	85	(13.9)	513	(83.8)	99	(16.2)	543	(88.7)	69	(11.3)
***Alcohol or drug use by husband***												
No	2333	(86.8)	355	(13.2)	2548	(94.8)	140	(5.2)	2592	(96.4)	96	(3.6)
Yes	1742	(83.0)	358	(17.1)	1832	(87.2)	268	(12.8)	1919	(91.4)	181	(8.6)
***Caste***												
General caste	2441	(85.5)	413	(14.5)	2587	(90.6)	267	(9.4)	2676	(93.8)	178	(6.2)
Other backward caste	1026	(87.0)	154	(13.1)	1087	(92.1)	93	(7.9)	1115	(94.5)	65	(5.5)
Scheduled tribe or caste	707	(81.1)	165	(18.9)	789	(90.5)	83	(9.5)	816	(93.6)	56	(6.4)
***Faith***												
Muslim	1544	(84.6)	282	(15.4)	1631	(89.3)	195	(10.7)	1687	(92.4)	139	(7.6)
Hindu	2465	(85.5)	417	(14.5)	2659	(92.3)	223	(7.7)	2742	(95.1)	140	(4.9)
Other	165	(83.3)	33	(16.7)	173	(87.4)	25	(12.6)	178	(89.9)	20	(10.1)
***Household socioeconomic quintile***												
1 Poorest	755	(77.9)	214	(22.1)	854	(88.1)	115	(11.9)	895	(92.4)	74	(7.6)
2	777	(83.0)	159	(17.0)	854	(91.2)	82	(8.8)	878	(93.8)	58	(6.2)
3	808	(86.5)	126	(13.5)	843	(90.3)	91	(9.7)	879	(94.1)	55	(5.9)
4	832	(89.2)	101	(10.8)	862	(92.4)	71	(7.6)	879	(94.2)	54	(5.8)
5 Least poor	833	(89.1)	102	(10.9)	869	(92.9)	66	(7.1)	890	(95.2)	45	(4.8)
**All (N)**	**4906**		**(100.0)**		**4906**		**(100.0)**		**4906**		**(100.0)**	

*Footnote*

SWEMWBS: Short Warwick-Edinburgh Mental Wellbeing Scale-7. PHQ-9: Patient Health Questionnaire-9. GAD-7: Generalised Anxiety Disorder-7

### Association between low wellbeing and symptoms of moderate-to-severe depression and anxiety

Table 3 shows outputs of logistic regression models for low wellbeing and reported symptoms of moderate-to-severe depression and moderate-to-severe anxiety, unadjusted and adjusted with socioeconomic and sociodemographic covariates. In the adjusted model, poorer and less educated women were more likely to report low wellbeing. Symptoms of anxiety or depression were more likely to be reported by widowed, separated or divorced women, women who were in paid employment and women who used drugs or alcohol themselves or whose husbands did. The findings were consistent with linear regression models using SWEMWBS, PHQ-9 and GAD-7 scores (Supplementary Table 1).

**Table 3:** Univariable and multivariable logistic regression models for low wellbeing and symptoms of moderate-to-severe depression and anxiety, by characteristics of 4906 ever-married women in urban informal settlements, Mumbai, India

Characteristic	Low mental wellbeing on SWEMWBS				Moderate-to-severe depression symptoms on PHQ-9				Moderate-to-severe anxiety symptoms on GAD-7			
	OR	[95% CI]	aOR	[95% CI]	OR	[95% CI]	aOR	[95% CI]	OR	[95% CI]	aOR	[95% CI]
***Marital status***												
Currently married	1				1		1		1		1	
Widowed, separated, divorced	1.2	[0.7, 2.0]	1.3	[0.6, 2.6]	6.0	[4.2, 8.6]	4.6	[2.6, 8.0]	5.2	[3.9, 7.0]	3.4	[1.8, 6.4]
***Age (years)***												
18–19	1				1		1		1		1	
20–29	1.0	[0.4, 2.9]	1.0	[0.4, 3.0]	0.9	[0.4, 2.2]	1.1	[0.4, 3.1]	0.7	[0.3, 1.9]	0.9	[0.3, 2.7]
30–39	0.9	[0.3, 2.8]	0.9	[0.3, 2.8]	0.9	[0.4, 2.3]	0.9	[0.3, 2.8]	0.8	[0.3, 2.1]	0.9	[0.3, 2.7]
40–49	0.8	[0.3, 2.4]	0.7	[0.2, 2.3]	1.5	[0.6, 3.9]	1.4	[0.5, 4.1]	1.4	[0.5, 3.7]	1.5	[0.5, 4.4]
***Schooling***												
No schooling	1		1		1		1		1		1	
Primary class 1–5	1.0	[0.8, 1.3]	1.1	[0.8, 1.4]	0.9	[0.6, 1.2]	0.9	[0.6, 1.2]	0.9	[0.6, 1.3]	0.8	[0.5, 1.2]
Middle 6–8	0.8	[0.6, 1.0]	0.8	[0.6, 1.0]	0.9	[0.7, 1.2]	0.9	[0.7, 1.3]	1.0	[0.7, 1.4]	1.0	[0.7, 1.4]
High 9–10	0.6	[0.5, 0.8]	0.7	[0.5, 0.8]	0.6	[0.4, 0.8]	0.7	[0.5, 1.0]	0.6	[0.4, 0.7]	0.7	[0.5, 0.9]
Higher 11–12	0.5	[0.4, 0.8]	0.5	[0.4, 0.8]	0.5	[0.3, 0.8]	0.8	[0.5, 1.3]	0.5	[0.3, 0.9]	0.7	[0.4, 1.3]
Above 12	0.5	[0.3, 0.7]	0.5	[0.4, 0.8]	0.4	[0.2, 0.6]	0.6	[0.3, 1.0]	0.4	[0.2, 0.7]	0.6	[0.3, 1.2]
***Employment of respondent***												
No	1		1		1		1		1		1	
Yes	0.9	[0.7, 1.1]	0.9	[0.7, 1.1]	2.0	[1.6, 2.5]	1.5	[1.2, 2.0]	2.0	[1.5, 2.5]	1.5	[1.1, 2.1]
***Employment of husband***												
No	1		1		1		1		1		1	
Yes	0.7	[0.4, 1.2]	0.8	[0.4, 1.5]	0.3	[0.2, 0.4]	0.7	[0.4, 1.3]	0.4	[0.2, 0.6]	1.0	[0.5, 2.0]
***Alcohol or drug use by respondent***												
No	1		1		1		1		1		1	
Yes	0.9	[0.7, 1.2]	0.7	[0.5, 1.0]	2.2	[1.7, 2.9]	1.4	[1.0, 1.9]	2.3	[1.6, 3.1]	1.4	[1.1, 1.9]
***Alcohol or drug use by husband***												
No	1		1		1		1		1		1	
Yes	1.4	[1.2, 1.6]	1.3	[1.1, 1.5]	2.7	[2.2, 3.3]	2.2	[1.8, 2.8]	2.6	[1.8, 3.6]	2.1	[1.4, 3.0]
***Caste***												
General caste	1		1		1		1		1		1	
OBC (Other backward caste)	0.9	[0.7, 1.1]	0.9	[0.7, 1.1]	0.8	[0.7, 1.1]	0.9	[0.7, 1.1]	0.9	[0.7, 1.2]	0.9	[0.7, 1.3]
ST/SC (Scheduled tribe or caste)	1.4	[1.1, 1.8]	1.5	[1.1, 1.9]	1.0	[0.8, 1.4]	0.9	[0.7, 1.3]	1.0	[0.7, 1.5]	1.0	[0.7, 1.4]
***Faith***												
Muslim	1		1		1		1		1		1	
Hindu	0.9	[0.7, 1.2]	1.0	[0.8, 1.3]	0.7	[0.6, 0.9]	0.8	[0.6, 1.0]	0.6	[0.5, 0.9]	0.6	[0.4, 0.9]
Other	1.1	[0.6, 1.8]	1.1	[0.6, 2.0]	1.2	[0.8, 1.9]	1.1	[0.6, 2.1]	1.4	[0.8, 2.2]	1.3	[0.7, 2.2]
***Household socioeconomic quintile***												
1 Poorest	1		1		1		1		1		1	
2	0.7	[0.6, 0.9]	0.7	[0.6, 0.9]	0.7	[0.5, 1.0]	0.7	[0.5, 1.0]	0.8	[0.6, 1.1]	0.8	[0.5, 1.2]
3	0.6	[0.4, 0.7]	0.6	[0.4, 0.8]	0.8	[0.6, 1.1]	0.8	[0.6, 1.1]	0.8	[0.5, 1.2]	0.8	[0.5, 1.2]
4	0.4	[0.3, 0.6]	0.5	[0.3, 0.6]	0.6	[0.5, 0.8]	0.6	[0.4, 0.8]	0.7	[0.5, 1.1]	0.7	[0.4, 1.0]
5 Least poor	0.4	[0.3, 0.6]	0.5	[0.4, 0.7]	0.6	[0.4, 0.8]	0.7	[0.4, 1.0]	0.6	[0.4, 0.9]	0.7	[0.4, 1.1]
**All (N)**	**4906**		**(100.0)**		**4906**		**(100.0)**		**4906**		**(100.0)**	

*Footnote*

SWEMWBS: Short Warwick-Edinburgh Mental Wellbeing Scale-7. PHQ-9: Patient Health Questionnaire-9. GAD-7: Generalised Anxiety Disorder-7. CI: confidence interval. OR: odds ratio. aOR: odds ratio adjusted with covariates for marital status, age, schooling, employment of respondent and husband, alcohol or drug use by respondent of husband, caste, faith and socioeconomic score

The findings from both unadjusted and adjusted logistic regression models for symptoms of moderate-to-severe depression or anxiety are summarized in Supplementary Table 2. Adjusting for background characteristics, the model suggested that women with low wellbeing had 2-fold and 2.2-fold greater odds of reporting symptoms of moderate-to-severe depression or moderate-to-severe anxiety than women with greater wellbeing.

## Discussion

In a survey of the mental health of almost 5000 women in urban informal settlements in Mumbai, 9% reported PHQ-9 scores suggesting depression, 6% GAD-7 scores suggesting anxiety and 15% SWEMWBS scores suggesting low wellbeing. The three sets of scores were mutually correlated. The number of women who reported low wellbeing exceeded the number who reported symptoms of anxiety or depression.

Prevalences of symptoms of moderate-to-severe depression and anxiety were lower in our study than reported in others in informal settlements in Mumbai or elsewhere in India. However, they were higher than the national average and higher than those reported by residents of non-slum areas [25, 26, 28–30, 69, 70]. Our findings may reflect population reality. It is conceivable that lower prevalence of anxiety and depression might reflect a greater sense of self-determination combined with improvements in environment, livelihoods and lifestyle in urban informal settlements. Migration, particularly from rural to urban centres, has been described as affecting the self-esteem of migrants positively as a result of economic opportunities and better livelihoods. One study found that rural migrants settled in urban regions have better later-life mental health [71]. Alternatively, the differences could be the result of methodological variation between studies in terms of sampling, scales or cut-offs for mental health conditions. It is possible that the stigma that accompanies mental health concerns led to underreporting of symptoms, although we presume that this would be a feature of all studies and our team of interviewers were well trained and supervised. With respect to mental wellbeing, the mean score (24.07) was actually higher than the mean score for women (23.59) in a national study in England [72].

Symptoms of anxiety, depression and low wellbeing were more likely to be reported by older women. Although we found a positive association between age and mental health concerns, our sample only included women under the age of 50. This means that we were unable to extend the analysis into older age, which is often characterized by a gradual decline in physical and mental health and an increased risk of disease [73]. It would be interesting to examine interaction between age and exposure to violence. Symptoms of anxiety or depression were more likely to be reported by widowed, separated or divorced women, women who were in remunerated occupations, and women who used drugs or alcohol themselves or whose husbands did. These findings are in line with the literature [2, 28, 59, 61, 64, 74–77]. Low wellbeing was more likely to be reported by older, poorer, and less educated women. A study from China suggested that the reductive effect of mental wellbeing in terms of happiness increased with age [21].

Paradoxically, use of alcohol or drugs was associated with fewer reports of low wellbeing. It is possible that this reflects their positive effects on tension and self-efficacy, in at least the short term [78], but we are wary of commenting on a single finding. Our analysis did not include exposure to intimate partner violence as an exposure covariate. However, it is well documented that women survivors are more likely to access mental healthcare services, and women with mental health conditions are more likely to experience intimate partner violence victimization [79].

Framing mental health along two continua corresponding with wellbeing and mental illness, we found some correspondence between low reported wellbeing and symptoms of anxiety and depression, and likewise between satisfactory wellbeing and absence of anxiety and depression. A range of studies have found similar associations [21, 80–83]. Our findings are in line with the WHO position on mental health [2], and with the idea of mental health as (at least) two continua. Perhaps the most interesting finding is the disjunction between the mental wellbeing and mental illness constructs. Women with low wellbeing did not necessarily report symptoms suggesting anxiety or depression, and women with anxiety or depression did not necessarily have low wellbeing. It seems feasible that individuals with mental health conditions can still enjoy mental wellbeing (or resist lower wellbeing) by having a support system, resilience, and style of coping [2, 84]. We have no studies in India with which to compare these findings because studies of anxiety and depression have not examined wellbeing, and vice versa, but the findings are in line with other studies suggesting that the possibility of mental wellbeing persists even when experiencing mental health conditions [85].

Our findings raise a number of questions for further exploration. Is it true that women are able to maintain a sense of wellbeing in the presence of potentially debilitating symptoms of anxiety or depression? If so, what is it that helps women exposed to multiple social determinants cope with mental illness to a degree to which their wellbeing is maintained? Will the findings differ for women in less challenging environments who are not exposed to poverty, insecurity of tenure and environmental degradation? Is it something to do with social and family support or cultural attitudes to the kinds of questions used in assessment scales? Would the findings be different for men? For example, in a study that administered the Positive and Negative Affect Schedule (PANAS)—Revised [86, 87] and the Satisfaction with Life Scale (SWLS) [88] to around 1000 adults in urban Bengaluru, subjective wellbeing was generally greater in men than in women [89].

### Limitations

The usual inferential limitations apply to our cross-sectional study. External validity is limited by the focus on a sample of ever-married women aged 18–49 years and their residence in urban informal settlements in India. The findings were based on self-reported answers to question scales which, though validated, may not be measuring quite what they seek to. We have no clinical diagnosis for constellations of symptoms suggestive of anxiety and depression. There is some concern that diagnostic categories and classification of mental disorders need revisiting in the context of global mental health [39]. Screening tools such as the PHQ-9 do not address cultural concepts of distress [90, 91]: ways in which cultural groups experience, understand, and communicate suffering, behavioural problems, or troubling thoughts and emotions [92]. Cultural concepts of distress now feature in the Diagnostic and Statistical Manual of Mental Disorders (DSM)-5 and could be included in approaches to screening [90].

### Clinical implications

Our study is the first in India to examine mental health along two continua and provides some support for the idea of doing so. It contributes to a growing body of work on wellbeing in South Asia and raises questions about the maintenance of wellbeing in the face of mental illness. That individuals experiencing mental illness can enjoy a state of wellbeing, lead meaningful lives and contribute to society is important and in agreement with India’s Mental Healthcare Act [36]. Conversely, that individuals with low wellbeing may not receive appropriate social and psychological support in the absence of diagnosable clinical conditions is of concern.

## Electronic supplementary material

Below is the link to the electronic supplementary material.

Supplementary Material 1

## Data Availability

Data can be accessed with permission at https://osf.io/szmup DOI 10.17605/OSF.IO/U3RGS.

## Abbreviations

CI: Confidence Interval
DSM-5: The Diagnostic and Statistical Manual of Mental Disorders-5
GAD-7: Generalised Anxiety Disorder-7
IEC: Institutional Ethics Committee
MHCA, 2017: Mental Healthcare Act, 2017
OR/aOR: Odds Ratio/ Adjusted Odds Ratio
PANAS-R: Positive and Negative Affect Schedule—Revised
PHQ-9: Patient Health Questionnaire-9
PUKAR: Partners for Urban Knowledge, Action, and Research
SNEHA: Society for Nutrition, Education and Health Action
SWEMWBS: Short Warwick-Edinburgh Mental Wellbeing Scale
SWLS: The Satisfaction with Life Scale
USD: United States dollar
TARA: Taking Action Reaching All
WEIRD: Western, Educated, Industrialized, Rich, and Democratic
WHO: The World Health Organization
