# Supplementary material for: Mental health on two continua: mental wellbeing and common mental disorders in a community-based cross-sectional study with women in urban informal settlements in India
Source: BMC Womens Health. 2024 Oct 9;24:555. doi: 10.1186/s12905-024-03389-1 (PMC11463130; doi:10.1186/s12905-024-03389-1)
Supplement: Supplementary file 1 — Supplementary Material 1 [file 12905_2024_3389_MOESM1_ESM.docx]

# Supplementary Tables

### Supplementary Table 1. Unadjusted and adjusted linear regression models for mental wellbeing, depression and anxiety, by characteristics of 4906 ever-married women in urban informal settlements, Mumbai, India.

|  | Mental wellbeing on SWEMWBS | | | | | | Moderate-to-severe depression symptoms on PHQ-9 | | | | | | Moderate-to-severe anxiety symptoms on GAD-7 | | | | | |
| --- | --- | --- | --- | --- | --- | --- | --- | --- | --- | --- | --- | --- | --- | --- | --- | --- | --- | --- |
|  | Unadjusted | | | Adjusted | | | Unadjusted | | | Adjusted | | | Unadjusted | | | Adjusted | | |
| **Characteristic** | Coef. | SE | [95% CI] | Coef. | SE | [95% CI] | Coef. | SE | [95% CI] | Coef. | SE | [95% CI] | Coef. | SE | [95% CI] | Coef. | SE | [95% CI] |
| ***Marital status*** |  |  |  |  |  |  |  |  |  |  |  |  |  |  |  |  |  |  |
| Currently married |  |  |  |  |  |  |  |  |  |  |  |  |  |  |  |  |  |  |
| Widowed, separated, divorced | -1.2 | 0.5 | [-2.1, -0.2] | -1.6 | 0.6 | [-2.9, -0.3] | 4.2 | 0.5 | [ 3.2, 5.2] | 4.6 | 0.9 | [2.7, 6.4] | 3.4 | 0.4 | [2.7, 4.2] | 3.3 | 0.7 | [2.0, 4.7] |
| ***Age (years)*** |  |  |  |  |  |  |  |  |  |  |  |  |  |  |  |  |  |  |
| 18-19 |  |  |  |  |  |  |  |  |  |  |  |  |  |  |  |  |  |  |
| 20-29 | 0.6 | 0.8 | [-1.0, 2.3] | 0.4 | 0.8 | [-1.2, 2.1] | -0.5 | 0.6 | [-1.6, 0.7] | -0.2 | 0.6 | [-1.4, 1.0] | -1.0 | 0.6 | [-2.2, 0.3] | -0.8 | 0.6 | [-1.9, 0.4] |
| 30-39 | 0.7 | 0.8 | [-1.0, 2.4] | 0.7 | 0.9 | [-1.1, 2.4] | -0.3 | 0.6 | [-1.5, 0.9] | -0.3 | 0.6 | [-1.4, 0.9] | -0.7 | 0.6 | [-2.0, 0.5] | -0.7 | 0.5 | [-1.8, 0.4] |
| 40-49 | 1.3 | 0.9 | [-0.5, 3.0] | 1.5 | 0.9 | [-0.3, 3.3] | 0.4 | 0.6 | [-0.8, 1.7] | 0.2 | 0.6 | [-1.0, 1.5] | -0.1 | 0.6 | [-1.3, 1.2] | -0.2 | 0.6 | [-1.3, 0.9] |
| ***Schooling*** |  |  |  |  |  |  |  |  |  |  |  |  |  |  |  |  |  |  |
| No schooling |  |  |  |  |  |  |  |  |  |  |  |  |  |  |  |  |  |  |
| Primary class 1-5 | 0.2 | 0.2 | [-0.2, 0.7] | 0.1 | 0.2 | [-0.4, 0.6] | -0.3 | 0.2 | [-0.7, 0.2] | -0.3 | 0.2 | [-0.7, 0.2] | -0.2 | 0.2 | [-0.6, 0.3] | -0.2 | 0.2 | [-0.6, 0.2] |
| Middle 6-8 | 0.9 | 0.3 | [ 0.4, 1.4] | 0.8 | 0.3 | [0.2, 1.3] | -0.2 | 0.2 | [-0.7, 0.2] | -0.1 | 0.2 | [-0.6, 0.3] | -0.1 | 0.2 | [-0.5, 0.2] | 0.0 | 0.2 | [-0.4, 0.3] |
| High 9-10 | 1.5 | 0.3 | [ 1.0, 2.1] | 1.3 | 0.3 | [0.8, 1.8] | -0.9 | 0.2 | [-1.3, -0.5] | -0.5 | 0.2 | [-0.9, -0.1] | -0.7 | 0.2 | [-1.0, -0.5] | -0.4 | 0.1 | [-0.7, -0.1] |
| Higher 11-12 | 2.1 | 0.4 | [ 1.3, 2.8] | 1.9 | 0.4 | [1.2, 2.7] | -1.2 | 0.3 | [-1.7, -0.6] | -0.5 | 0.3 | [-1.1, 0.1] | -1.1 | 0.2 | [-1.6, -0.7] | -0.6 | 0.2 | [-1.0, -0.2] |
| Above 12 | 2.7 | 0.4 | [ 2.0, 3.5] | 2.5 | 0.4 | [1.8, 3.2] | -1.6 | 0.3 | [-2.1, -1.0] | -0.8 | 0.3 | [-1.3, -0.3] | -1.3 | 0.2 | [-1.8, -0.8] | -0.6 | 0.2 | [-1.0, -0.2] |
| ***Employment of respondent*** |  |  |  |  |  |  |  |  |  |  |  |  |  |  |  |  |  |  |
| No |  |  |  |  |  |  |  |  |  |  |  |  |  |  |  |  |  |  |
| Yes | -0.1 | 0.2 | [-0.4, 0.3] | 0.0 | 0.2 | [-0.3, 0.4] | 1.3 | 0.2 | [ 1.0, 1.6] | 0.8 | 0.1 | [0.5, 1.1] | 1.1 | 0.1 | [ 0.8, 1.4] | 0.7 | 0.1 | [0.4, 1.0] |
| ***Employment of husband*** | |  |  |  |  |  |  |  |  |  |  |  |  |  |  |  |  |  |
| No |  |  |  |  |  |  |  |  |  |  |  |  |  |  |  |  |  |  |
| Yes | 1.3 | 0.5 | [ 0.2, 2.4] | 0.7 | 0.5 | [-0.4, 1.8] | -3.5 | 0.6 | [-4.8, -2.3] | -1.7 | 0.7 | [-3.1, -0.2] | -2.8 | 0.6 | [-3.9, -1.6] | -1.3 | 0.6 | [-2.5, 0.0] |
| ***Alcohol or drug use by respondent*** | |  |  |  |  |  |  |  |  |  |  |  |  |  |  |  |  |  |
| No |  |  |  |  |  |  |  |  |  |  |  |  |  |  |  |  |  |  |
| Yes | -0.2 | 0.3 | [-0.7, 0.4] | 0.5 | 0.3 | [-0.1, 1.1] | 1.6 | 0.3 | [ 1.0, 2.1] | 0.6 | 0.2 | [0.1, 1.0] | 1.3 | 0.2 | [ 0.9, 1.8] | 0.5 | 0.2 | [0.2, 0.9] |
| ***Alcohol or drug use by husband*** | |  |  |  |  |  |  |  |  |  |  |  |  |  |  |  |  |  |
| No |  |  |  |  |  |  |  |  |  |  |  |  |  |  |  |  |  |  |
| Yes | -1 | 0.2 | [-1.3, -0.6] | -0.6 | 0.2 | [-1.0, -0.3] | 1.6 | 0.2 | [ 1.3, 2.0] | 1.2 | 0.2 | [0.9, 1.6] | 1.3 | 0.2 | [ 1.0, 1.6] | 1.0 | 0.1 | [0.7, 1.3] |
| ***Caste*** |  |  |  |  |  |  |  |  |  |  |  |  |  |  |  |  |  |  |
| General caste |  |  |  |  |  |  |  |  |  |  |  |  |  |  |  |  |  |  |
| OBC (Other backward caste) | -0.2 | 0.3 | [-0.7, 0.3] | -0.2 | 0.2 | [-0.6, 0.2] | -0.1 | 0.2 | [-0.4, 0.2] | 0.0 | 0.1 | [-0.3, 0.2] | -0.1 | 0.1 | [ -0.3, 0.1] | 0.0 | 0.1 | [-0.2, 0.2] |
| ST/SC (Scheduled tribe or caste) | -0.6 | 0.3 | [-1.2, 0.0] | -0.6 | 0.2 | [-1.1, -0.1] | 0.3 | 0.2 | [-0.1, 0.6] | 0.1 | 0.2 | [-0.2, 0.4] | 0.1 | 0.2 | [ -0.2, 0.5] | 0.0 | 0.1 | [-0.3, 0.2] |
| ***Faith*** |  |  |  |  |  |  |  |  |  |  |  |  |  |  |  |  |  |  |
| Muslim |  |  |  |  |  |  |  |  |  |  |  |  |  |  |  |  |  |  |
| Hindu | 0.4 | 0.4 | [-0.5, 1.3] | 0.0 | 0.4 | [-0.8, 0.7] | -0.6 | 0.1 | [-0.9, -0.4] | -0.5 | 0.2 | [-0.8, -0.2] | -0.6 | 0.1 | [-0.9, -0.3] | -0.5 | 0.1 | [-0.8, -0.2] |
| Other | 0.7 | 0.7 | [-0.6, 2.0] | 0.6 | 0.7 | [-0.7, 2.0] | 0.6 | 0.3 | [ 0.0, 1.3] | 0.3 | 0.4 | [-0.5, 1.1] | 0.3 | 0.2 | [-0.2, 0.8] | 0.1 | 0.3 | [-0.4, 0.6] |
| ***Household socioeconomic quintile*** | |  |  |  |  |  |  |  |  |  |  |  |  |  |  |  |  |  |
| 1 Poorest |  |  |  |  |  |  |  |  |  |  |  |  |  |  |  |  |  |  |
| 2 | 1.1 | 0.2 | [ 0.6, 1.6] | 1.0 | 0.2 | [0.5, 1.4] | -0.6 | 0.2 | [-1.0, -0.3] | -0.6 | 0.2 | [-1.0, -0.3] | -0.5 | 0.2 | [-0.9, -0.1] | -0.5 | 0.2 | [-0.8, -0.1] |
| 3 | 1.9 | 0.3 | [ 1.2, 2.6] | 1.7 | 0.3 | [1.0, 2.3] | -0.6 | 0.2 | [-1.1, -0.2] | -0.6 | 0.2 | [-1.0, -0.1] | -0.4 | 0.2 | [-0.8, 0.0] | -0.3 | 0.2 | [-0.7, 0.1] |
| 4 | 2.2 | 0.3 | [ 1.5, 2.9] | 1.8 | 0.3 | [1.2, 2.5] | -0.9 | 0.2 | [-1.3, -0.5] | -0.9 | 0.2 | [-1.3, -0.5] | -0.5 | 0.2 | [-0.9, -0.1] | -0.5 | 0.2 | [-0.9, -0.1] |
| 5 Least poor | 3.2 | 0.3 | [ 2.5, 3.9] | 2.5 | 0.3 | [1.9, 3.2] | -1.3 | 0.2 | [-1.7, -0.8] | -1.0 | 0.2 | [-1.5, -0.5] | -1.0 | 0.2 | [-1.4, -0.6] | -0.7 | 0.2 | [-1.1, -0.3] |

*Footnote*

SWEMWBS: Short Warwick-Edinburgh Mental Wellbeing Scale-7. PHQ-9: Patient Health Questionnaire-9. GAD-7: Generalised Anxiety Disorder-7. CI: confidence interval. Coef: coefficient. SE: standard error. CI: confidence interval. Ref: reference category. Coefficient adjusted with covariates for marital status, age, schooling, employment of respondent and husband, alcohol or drug use by respondent of husband, caste, faith and socioeconomic score.

### Supplementary Table 2. Univariable and multivariable logistic regression models for low wellbeing and symptoms of moderate-to-severe depression and anxiety, for 4906 ever-married women in urban informal settlements, Mumbai, India.

| **Characteristic** | Moderate-to-severe depression symptoms on PHQ-9 | | | | Moderate-to-severe anxiety symptoms on GAD-7 | | | |
| --- | --- | --- | --- | --- | --- | --- | --- | --- |
|  | OR | [95% CI] | aOR | [95% CI] | aOR | [95% CI] | aOR | [95% CI] |
|  |  |  |  |  |  |  |  |  |
| ***Low-wellbeing*** |  |  |  |  |  |  |  |  |
| No | 1 |  | 1 |  | 1 |  |  |  |
| Yes | 2.1 | [1.6, 2.8] | 2.0 | [1.4, 2.6] | 2.4 | [ 1.7, 3.4] | 2.2 | [1.5, 3.2] |
| **Background** |  |  |  |  |  |  |  |  |
| ***Marital status*** |  |  |  |  |  |  |  |  |
| Currently married | 1 |  | 1 |  | 1 |  | 1 |  |
| Widowed, separated, divorced | 6.0 | [4.2, 8.6] | 1.1 | [0.5, 2.2] | 5.2 | [3.9, 7.0] | 1.1 | [0.5, 2.3] |
| ***Age (years)*** |  |  |  |  |  |  |  |  |
| 18-19 | 1 |  |  |  | 1 |  |  |  |
| 20-29 | 0.9 | [0.4, 2.2] | 1.0 | [0.4, 3.0] | 0.7 | [0.3, 1.9] | 1.0 | [0.4, 2.9] |
| 30-39 | 0.9 | [0.4, 2.3] | 0.9 | [0.3, 2.8] | 0.8 | [0.3, 2.1] | 0.9 | [0.3, 2.7] |
| 40-49 | 1.5 | [0.6, 3.9] | 0.7 | [0.2, 2.2] | 1.4 | [0.5, 3.7] | 0.7 | [0.2, 2.1] |
| ***Schooling*** |  |  |  |  |  |  |  |  |
| No education | 1 |  | 1 |  | 1 |  | 1.0 |  |
| Primary class 1-5 | 0.9 | [0.6, 1.2] | 1.1 | [0.8, 1.4] | 0.9 | [0.6, 1.3] | 1.1 | [0.8, 1.4] |
| Middle 6-8 | 0.9 | [0.7, 1.2] | 0.8 | [0.6, 1.1] | 1.0 | [0.7, 1.4] | 0.8 | [0.6, 1.0] |
| High 9-10 | 0.6 | [0.4, 0.8] | 0.7 | [0.5, 0.9] | 0.6 | [0.4, 0.7] | 0.7 | [0.5, 0.9] |
| Higher 11-12 | 0.5 | [0.3, 0.8] | 0.5 | [0.4, 0.8] | 0.5 | [0.3, 0.9] | 0.5 | [0.4, 0.8] |
| Above 12 | 0.4 | [0.2, 0.6] | 0.6 | [0.4, 0.8] | 0.4 | [0.2, 0.7] | 0.5 | [0.4, 0.8] |
| ***Employment of respondent*** |  |  |  |  |  |  |  |  |
| No | 1 |  | 1 |  | 1 |  | 1 |  |
| Yes | 2.0 | [1.6, 2.5] | 0.8 | [0.7, 1.0] | 2.0 | [1.5, 2.5] | 0.8 | [0.7, 1.1] |
| ***Employment of husband*** |  |  |  |  |  |  |  |  |
| No | 1 |  |  |  | 1 |  |  |  |
| Yes | 0.3 | [0.2, 0.4] | 0.8 | [0.5, 1.6] | 0.4 | [0.2, 0.6] | 0.8 | [0.4, 1.5] |
| ***Alcohol or drug use by respondent*** |  |  |  |  |  |  |  |  |
| No | 1 |  | 1 |  | 1 |  | 1 |  |
| Yes | 2.2 | [1.7, 2.9] | 0.7 | [0.5, 1.0] | 2.3 | [1.6, 3.1] | 0.7 | [0.5, 1.0] |
| ***Alcohol or drug use by husband*** |  |  |  |  |  |  |  |  |
| No | 1 |  | 1 |  | 1 |  | 1 |  |
| Yes | 2.7 | [2.2, 3.3] | 1.2 | [1.0, 1.4] | 2.6 | [1.8, 3.6] | 1.2 | [1.0, 1.4] |
| ***Caste*** |  |  |  |  |  |  |  |  |
| General caste | 1 |  | 1 |  | 1 |  | 1 |  |
| OBC (Other backward caste) | 0.8 | [0.7, 1.1] | 0.9 | [0.7, 1.2] | 0.9 | [0.7, 1.2] | 0.9 | [0.7, 1.2] |
| ST/SC (Scheduled tribe or caste) | 1.0 | [0.8, 1.4] | 1.5 | [1.1, 1.9] | 1.0 | [0.7, 1.5] | 1.5 | [1.1, 1.9] |
| ***Faith*** |  |  |  |  |  |  |  |  |
| Muslim | 1 |  | 1 |  | 1 |  | 1 |  |
| Hindu | 0.7 | [0.6, 0.9] | 1.0 | [0.8, 1.4] | 0.6 | [0.5, 0.9] | 1.0 | [0.8, 1.4] |
| Other | 1.2 | [0.8, 1.9] | 1.1 | [0.6, 2.0] | 1.4 | [0.8, 2.2] | 1.1 | [0.6, 2.0] |
| ***Household socioeconomic quintile*** |  |  |  |  |  |  |  |  |
| 1 Poorest | 1 |  | 1 |  | 1 |  | 1 |  |
| 2 | 0.7 | [0.5, 1.0] | 0.7 | [0.6, 0.9] | 0.8 | [0.6, 1.1] | 0.7 | [0.6, 0.9] |
| 3 | 0.8 | [0.6, 1.1] | 0.6 | [0.4, 0.8] | 0.8 | [0.5, 1.2] | 0.6 | [0.4, 0.8] |
| 4 | 0.6 | [0.5, 0.8] | 0.5 | [0.3, 0.7] | 0.7 | [0.5, 1.1] | 0.5 | [0.3, 0.7] |
| 5 Least poor | 0.6 | [0.4, 0.8] | 0.5 | [0.4, 0.7] | 0.6 | [0.4, 0.9] | 0.5 | [0.4, 0.7] |
| **All (N)** | **4906** | | **(100.0)** | | **4906** | | **(100.0)** | |

*Footnote*

SWEMWBS: Short Warwick-Edinburgh Mental Wellbeing Scale-7. PHQ-9: Patient Health Questionnaire-9. GAD-7: Generalised Anxiety Disorder-7. CI: confidence interval. OR: odds ratio. aOR: odds ratio adjusted with covariates for marital status, age, schooling, employment of respondent and husband, alcohol or drug use by respondent of husband, caste, faith and socioeconomic score.
